# On Thrush in Children

**Published:** 1847-10

**Authors:** 


### Art. XII.

*Om Torsk hos Barn.* Af Dr. Fr. Th. Berg, Ofverläkare vid Stockholms Allm. Barnhus, &c.

*On Thrush in Children.* By Dr. Fr. Th. Berg, Physician in Chief to the General Children's Hospital in Stockholm, &c.—*Stockholm*, 1846. 8vo, pp. 157.

We have in former notices of the labours of our Swedish brethren, awarded, we hope, due praise to their steady pursuit of the legitimate objects of medical science; the work now before us, is a further proof, that we have not overvalued their merits. Dr. Berg's modest little volume has afforded us great satisfaction. We have admired the diligence and perseverance requisite for so many and such varied experiments and observations, while the candour and good faith with which these researches are recorded is a guarantee for their perfect accuracy.

It is true that the malady of which our author treats is regarded by many, as a disease of no great importance in a practical point of view; but those who have experienced the difficulty of finding appropriate remedies to check its progress, will be glad to acquire a more intimate knowledge of a disorder too lightly noticed in works on infantile disease. To the scientific investigator this little essay will, we are sure, be valuable from the numerous and well conducted experiments it records, while to the microscopic observer its interest is enhanced by the fresh paths of research which it lays open for the still further cultivation of pathological and physiological subjects.

*Definition.* The following definition of Thrush by our author differs widely from that of earlier writers.

"Thrush is a deposit upon the mucous membrane, in the form of small points, rings, conical or hemispherical elevations; at other times it covers large surfaces with a continuous membrane. It is at first of a milk-white or pearly hue, but occasionally when undisturbed, or when it affects children at the breast, it assumes a gray or yellowish colour, but it rarely becomes darker without the addition of foreign matters. It is more or less of a soft cheesy consistence; and in thickness varies from that of the thinnest paper to half a line or more. At first it adheres pretty closely to the parts on which it appears, but later, it becomes more loose, and at length is spontaneously detached without any lesion of continuity of the subjacent mucous membrane. Thrush is met with most frequently on the edges of the lips, where the skin merges into the mucous membrane, and on the inside of the cheeks, on the gums, the hard palate, and the upper and under surfaces of the tongue; as likewise in the fauces and œsophagus, down to the cardiac orifice of the stomach."

*Microscopical character.* The great object of the author is to elucidate the microscopical character of the disease. We make no apology here for translating the writer's own words, as it would not be possible to condense his descriptions without materially impairing their completeness. In microscopical anatomy the omission of one small part of the appearances observed is hardly less injurious, than the leaving out of an acid or an alkali in a chemical experiment.

"The white coating consists of epithelium thickened by the swelling of its constituent cells, and from this epithelium there springs a parasitic fungus in greater or less quantity, so that the chief portion of a patch of aphthæ is composed either of epithelium, or else of the parasitic growth. Now the relative proportions of these two substances seem to depend upon the length of time that has elapsed since the growth of the parasite commenced, which varies in different children, and it is also in relation to the density of the epithelial thickening. More or less of molecular albuminous matter is also to be found in these patches.

"When the parasitic growth and the epithelial condensation is confined merely to the extremities of the smaller papillæ, (as at the point of the tongue) they have the appearance of small isolated specks, but when they appear upon portions of the mucous membrane where the papillæ are less prominent and where the intervals between these are filled by a denser epithelium, (such as is seen on all parts of the mucous membrane of the mouth save on the upper surface of the tongue,) the white coating then assumes those forms of circles, of interlacing bands, or of hemispherical elevations, so frequently observed in parasitic vegetations, where they are permitted to increase freely without any mechanical obstacle to their growth. Lastly, when both the epithelial and the parasitic growths alike proceed vigorously, those spots which at first were isolated, coalesce more and more into a continuous covering, the cohesion of which is maintained not merely by the natural adhesion of the epithelial cells, but also by the interlacing of the parasitic fibres, among themselves, and between the cells of the epithelium." (p. 3.)

*Colour.* The alterations of colour observed in thrush and aphthæ are next described at full length, and the cause of these variations is carefully explained.

The usual colour of aphthæ is well known to be white or pearl gray, but when the parasitic growth increases and forms numerous sporuli on the surface, the spots tend to assume a yellow or greenish hue, like other fungoid growths where sporuli are produced in abundance. In this way we explain the fact that aphthæ occurring in children brought up at the breast and in their own homes, are usually thinly scattered, and of the usual white or gray appearance; while in lying-in hospitals, where children rarely get the breast, where their mouths are seldom cleansed during their prolonged slumbers, the aphthous vegetations advance unchecked, and constantly assume the yellow or green colour.

*Attachment.* Aphthæ are well known to be more firmly adherent in some parts than in others, and to vary in this respect at different periods of their existence. Our author accounts for these differences in the following paragraphs.

"The fine threads or fibres of the parasite insert themselves in every direction amid the superior layer of epithelial cells, and, according to Gruby they pass even into the cells themselves. By this means they acquire a firm adhesion to the neighbouring parts, and involve many epithelial cells, among their meshes. This will explain why aphthæ are so firmly attached when first formed, and will account also for their varying degrees of adhesion in different parts. Certain surfaces present numerous papillæ, and these latter are often cloven at the summit in many directions, thus affording an innumerable quantity of furrows and depressions for receiving the fibres of the parasitic growth. In this way aphthæ become firmly fixed upon the tongue in spite of the perpetual movement of that organ, while on other parts of the buccal mucous membrane, where the papillæ are fewer, smaller, and less elevated, and rarely cloven at the summit, they adhere with much less tenacity.

It will be known however to the reader that these vegetations do not sink deeper than the epithelial layer and that this layer is constantly renewed by the destruction of its most external cells, their place being supplied by new cells from the inferior surface. The spread of the parasitic fibres among the cells of the epithelium will tend still further to loosen the adhesion of the latter. But even these causes combined are scarcely sufficient to account for the whole process of softening and throwing off of the aphthous patches; it seems that the same (*undiscovered*) cause which renders the epithelium the appropriate soil for the growth of these parasites, produces also in its cells an operation similar to that of maceration if we may judge from the white colour assumed under such circumstances by the membrane. In consequence of this, there ensues, sooner or later, the throwing off of the whole aphthous patch with the subjacent epithelium, after which there remains the mucous membrane covered with the thinnest possible layer of recently formed epithelium; and the thinner the latter is, the deeper is the hue of the subjacent mucous membrane.

"Aphthous vegetations therefore require to have a firm root among the cells of the epithelium, before they can vegetate upon those surfaces where there is a constant motion of the parts. Consequently the epithelium on which we find numerous ciliæ in constant movement is eminently unfitted for the localization of aphthæ, nor will the epithelium which is composed of a single layer of cells be better adapted to this purpose, as the membrane will then be undergoing constant and rapid change. We must therefore conclude that the best *soil* for the growth of aphthous vegetations is undoubtedly that portion of the epithelium where the absence of ciliæ gives full scope to the implantation of sporules, and where several layers of cells afford full room for the spread and interlacing of the vegetable fibres, and where, finally, the membrane being less rapidly changed, due time is allowed for the development of the vegetable growth. Such is the character of the epithelial covering from the beginning of the mucous membrane on the free edge of the lips, throughout the whole cavity of the mouth, the fauces, and œsophagus, down to the cardia, while the adhesion of the parasitic growth is greatly favoured on this surface by the numerous furrows lying between the thickly strown papillæ, and by the cloven summits of the latter as they are seen on the upper surface of the tongue.

This species of epithelial covering ceases however at the mouths of the salivary ducts, which are lined with a single layer of *cylinder epithelium*; it terminates likewise at the upper and back part of the velum palati; at the mouths of the Eustachian tubes, and at the base too of the epiglottis, immediately above the upper edge of the superior ligamenta glottidis. In all these points it is replaced by ciliated epithelium, while at the cardiac orifice of the stomach it gives place to a single layer of *cylinder-epithelium*" (pp. 3-5.)

*Habitat.* Dr. Berg questions the truth of the assertions of various authors that true aphthæ have been found in the respiratory organs or in the stomach. Most of those that maintain this opinion have not, he observes, examined the appearances they describe with the microscope, and without the aid of this instrument, he is unwilling to accept of their diagnosis. On the other hand, our author observes that nothing is more easy than for a small portion of aphthous matter to escape from the œsophagus into the stomach, or into the respiratory organ. He candidly acknowledges that in 1842, he himself announced the discovery of aphthæ firmly adhering to the mucous membrane of the stomach, but since that period he has never been able to detect such appearances, and he is now strongly inclined to doubt the accuracy of his former opinion.

*Microscopical character.* Our author again passes to the microscopical character of aphthæ. The powers to which he subjected these appearances for examination increased the objects by 200 to 300 diameters.

"Under a microscope of the above powers we find," says he, "that an aphthous crust consists of epithelial cells, with a more or less interweaved web of fibres, and a variable number of spherical or oval cells, without any sign of exudation-corpuscles, but only a small quantity of molecular albuminous deposit. These cells are, with the exception of the nucleus, everywhere transparent, and have a sharply-defined edge, they vary in size according as they are oval, spherical, or more prolonged (0·0004 — 0·0015 m. and more). Sometimes they present no nucleus, or an almost imperceptible trace thereof, but the larger ones contain almost always nucleated cellules, sometimes two or even more. These nuclei lie usually in the centre, but very frequently also at the end of the cells. We can often trace the successive development of these cells from a spherical one of the smallest size to an oval cell, and thence to a filament; and we have no doubt ourselves that the smaller cells are sporules, out of whose development the larger oval cells are formed, and finally, the filaments in the same manner as has been observed in other fungoid growths of this nature. The sporules above noticed bear a strong analogy to those of yeast (Torula cerevisiæ), and this holds good not merely as regards their external form, but likewise in their power of resisting chemical reagents." (pp. 7-8.)

Numerous projecting fibrils are observed on the circumference of an aphthous crust when submitted to the microscope, but these are rendered infinitely more clear by a weak solution of potash, which dissolves the albumen, and renders the cells of the epithelium transparent, while, at the same time, it diminishes their intimate cohesion, and the network of vegetable fibres is more plainly seen.

"These fibres are cylindrical, with sharply-defined dark edges, and their centres are transparent in transmitted light; they are generally equal in thickness, but at times they are, as it were, knotted together, and divided by distinct walls of separation (skiljovägg). Their diameter is from 0·001 m. to 0·004 m. and more; their length from 0·1 m. — 0·2 m. If the dividing walls are sharply defined, and lie near to each other, the fibrils take on the aspect of a string of beads, and occasionally they closely resemble the tubes formed in higher organizations out of cylindrical cells. In their interior, these fibrils often exhibit nucleated cells; occasionally these are very numerous, and of small size, but at times they are larger. In their course the fibrils divide into numerous branches, whose diameter is not less than that of the original stem, and I have occasionally observed these ramifications to increase in thickness, at their free extremity, and to terminate in a club-shaped end with a species of cell. From the sides of the fibrils spring numerous sporules forming a point of departure for new ramifications.

"These fibrils are, it seems, as little affected by chemical re-agents as the cells we have before described.

"Careful investigation has shown us that these cells are placed upon the sides of the fibrils, and in particular that they are congregated around the terminations of the latter. It must therefore be admitted that the cells and the fibrils are both constituent parts of one and the same organization. Where this growth vegetates undisturbed, its fibrils penetrate between the layers of the epithelial cells, but do not extend deeper than the inferior layer, though they spread laterally in every direction. On the free surface of the epithelium, the ramifications rise above the surface, exhibiting at the same time an abundant fructification, which gives a yellowish hue to the exterior." (pp. 8-9.)

*Thrush a parasite.* Dr. Berg considers it to be perfectly established that the parasitic growth just described is a constituent part of thrush, for the latter, he observes, is never met with without the parasite, which is to be seen at the very earliest appearance of the aphthæ, and continues present throughout the whole of their existence on the mucous membrane. "Indeed," continues he, "I do not consider those affections to be aphthous where this parasitic growth is not to be found; and lastly, I advise all who think they have observed true aphthæ without its parasitic constituent, to add a little caustic potass to the supposed aphthæ before they determine on its nature."

It is impossible indeed, according to our author, to explain the nature and course of this disease if we deny its parasitic character.

*Conditions of life of the parasite.* The next question discussed by Dr. Berg is the important one, whether the parasitic fungus can vegetate only on the living mucous membrane, or whether it can be produced and grow in other menstrua, and out of the human body. Many carefully conducted experiments, occupying some pages of the work, are here recorded to decide this point. We have only room for the conclusions arrived at by our author, and which he sums up as follows:

"1. The aphthous parasite can propagate itself in appropriate menstrua out of the body, and this not only when the aphthous crust is mingled with various animal fluids, but also when completely separated and cleansed from these.

"2. Its growth in such cases proceeds not only in a temperature equal to that of the human body, but likewise in one that is much lower.

"3. Aphthæ seem to require for their growth the presence of a body containing azote, such as albumen, as also that of materials for the generation of acid.

"4. Out of the body aphthæ seem to develop themselves in two different forms, either in that of a great preponderance of sporules (sporidier), when a white filmy membrane forms on the surface of the fluid; or again they appear chiefly as stems ramifying through the fluid, or aggregated into a felt-like mass.

"A solution of potass will always dissolve the molecular deposit of albumen, leaving the fibres and cells of the parasite totally unchanged." (p. 14.)

Dr. Berg admits that the parasitic growth may be propagated in bodies previously unaffected, by sporules floating in the atmosphere, particularly under certain *electro-chemical* conditions of the air, and also of the bodies they affect.

*Description of the disease.* The disease in question is next described by Dr. Berg. As his theory of the malady differs widely from that of most pathologists, excepting a few of the most recent date, so we find his picture of the disorder, to present many discrepancies from the generally received descriptions. This was only what might be expected, when a totally new character is given to an affection of very common occurrence. It remains for the medical world to pass judgment on the parasitic doctrine of disease. We are however on one point fully satisfied, that should this theory, as we confidently believe, turn out to be in all respects well founded, the work of Dr. Berg will ever be a standard book with microscopical observers, and, like the observations of Leuwenhoek, will not lose its value amid the improved researches of future ages.

Our author distinguishes aphthæ into *discrete* and *confluent*, and likewise calls our attention to the different portions of the body in which it is found. He has not himself seen the redness and increased heat of the mouth which is stated by most observers to precede the outbreak of aphthæ, and he therefore does not recognise stomatitis as its necessary or even as its frequent antecedent. He does not however deny the possibility of thrush appearing on the inflamed mucous membrane, but he regards it then as a complication of the primary disease. The two varieties of stomatitis, *S. vesicularis* and *S. folliculosa*, have been too often described as true aphthous maladies, but Dr. Berg insists on the fact that these two forms, however severe they may be, never present the parasitic growth, characteristic of true thrush. It is this error which has given rise to the names of ulcers and pustular aphthæ. The question whether severe thrush ever appears without general disorder of the constitution, is answered by our author in the affirmative, but he owns that general symptoms are more frequently present. Those however which by medical writers are described as peculiar to thrush, are common, he thinks, to many of the other disorders of children.

The general torpor and great tendency to sleep, so often observed before a severe invasion of the malady, may, he thinks, act as a mechanical predisposing cause, by allowing the mouth to remain so long in a quiescent state, and thus favouring the rapid development of the parasitic growth. But Dr. Berg believes thrush mainly to depend on a "bio-chemical alteration of the fluids," and especially of the secretions of the buccal cavity. These secretions he has generally found to be acid, though on a few occasions they gave an alkaline reaction; but when thrush had decidedly appeared the secretions of the mouth were invariably strongly acid. Much of the disorder of digestion may, our author suggests, arise from the quantity of loosened aphthous crusts swallowed by the patient, but we do not ourselves believe that they can be sufficiently abundant to produce this effect.

Out of 115 patients affected with thrush in the Lying-in Hospital of Stockholm in the year 1845, only 29 had stools of the normal yellow colour throughout the whole course of the disease, while in the remaining 86 green evacuations appeared simultaneously with the invasion of the disease, or supervened at a later period of the malady. There seems therefore, to be some connexion between the green stools and the presence of thrush on the mucous membrane; and to the investigation of this relation our author accordingly proceeds.

In general the colic, vomiting, and diarrhea indicating disorder of the digestive organs, are in a direct relation to the extent and severity of the aphthous affection. The green evacuations are generally acid according to Dr. Berg, and he believes this peculiar hue of the fæces to be caused by an excessive proportion of acid, or by the presence of acids which do not normally exist in the digestive tube or at least are found there in exceedingly small quantities. Our author suggests that this excess of acidity may, in part, be occasioned by the admixture of fragments of the parasitic growth with the contents of the stomach, and he hints that the action thus induced is somewhat analogous to the process of fermentation from yeast. To elucidate and confirm these suppositions Dr. Berg has carefully performed a great number of experiments, the recital of which occupies five or six pages of his essay. We cannot of course in this brief notice give any of these experiments in detail, but they appear to us to have been scientifically conducted, and the instances where the results have been contrary to the author's expectations are candidly recorded. The conclusions he draws from these experiments are as follows.

"1. Aphthæ, whether attached to their original seat, or when loose and floating in a solution of starch, of sugar, or of sugar of milk, may cause the formation of lactic acid in a temperature from + 12° to — 15° Centigrade (79° to 30° Fahr.)

"2. Aphthæ have the property of throwing down albumen, and of forming casein, out of the aforementioned solutions, probably by the generation of acid.

"3. These operations take place much more rapidly in a temperature corresponding to that of the human body.

"4. The growth of the aphthous parasite in a solution of sugar containing albumen, and at the temperature of the human body, is accompanied with a *fermentation-like* development of carbonic acid gas, and with the formation of lactic acid. Under certain circumstances, acetic acid is produced by the influence of aphthæ.

"5. Yeast likewise produces the formation of acid in the above solutions, when it does not occasion fermentation, and the acid thus formed is probably the lactic, but yeast is much slower in producing this effect.

"6. Although it must be acknowledged that the coats of the stomach, and animal membrane in general, nay, even that albumen alone, may change a solution of sugar into lactic acid, and that thus the other constituents of the aphthous parasite, i. e., the molecular albuminous deposit, the epithelium, and portions of lactic acid may contribute to the generation of acid, yet it cannot be denied that the chief influence of aphthæ, in this respect, depends on the presence of the parasitic growth itself." (pp. 36-7.)

A great difference was found in the course of these experiments between the action of the reagents on cow's milk, and on that secreted by the human female. Dr. Berg regards this as a fact of great importance in reference to the question of bringing up children by hand, instead of their deriving their nourishment from its natural source, the mother's breast; and as affording a partial explanation of the greater frequency of thrush in the one case, than in the other.

We would gladly have continued our extracts from this most interesting portion of Dr. Berg's work, but want of space obliges us to refrain. After tracing the production of acidity in the primæ viæ through the agency of the aphthous vegetations, he proceeds to consider its direct influence on the functions of digestion and nutrition. Dr. Berg inclines to the belief that the parasitic growth may vegetate without any fixed seat, when mingled with the fluid contents of the stomach, and it will thus tend to increase still further the excess of acid. Finally, our author repeats his conviction that both the local and the general symptoms which accompany thrush in the child are, in most cases, immediate or secondary consequences of the presence of the parasite, and are not to be regarded as the causes of that fungoid vegetation. If thrush were only a plastic deposit, the result of inflammatory action, we should find it in most cases preceded by symptoms of general disorder or of local inflammation, both of which are constantly wanting.

Our author does not regard the mildness or severity of the symptoms as dependent upon any variation in the nature or specific character of the parasite, but as entirely influenced by the part of the body in which it occurs; by the idiosyncrasy of the individual; and, lastly, by external circumstances—as bad food, deficient ventilation, and want of attendance.

*The course of the disease,* is divided by Dr. Berg into the following stages:—1. To the first he gives the expressive name of "*Inplantations-stadium,*" and no English word perhaps could so well express the period when the first germination of the parasite is taking place upon the mucous membrane. 2. The stage of local increase of the parasite. 3. The period of the secondary general symptoms, i.e. the excessive production of acidity in the primæ viæ, &c., as before described. 4. The stage of convalescence. Of these four stages the second is the only one which is constantly present. An interval of from twenty-four hours to seven or eight days is often observed between the second and the commencement of the third stage.

"If," the author continues, "it may be conceded that the general symptoms of the third stage arise from the increase of sporules of the parasite in the liquid contents of the intestinal canal, attended with the development of carbonic acid gas, we can easily comprehend the necessity of a brief interval between the appearance of the vegetation on the tongue and the commencement of the fermentation (?) process in the intestinal canal, during which time a sufficient quantity of sporules have been swallowed to produce a competent catalytical operation on the contents of the intestines." (p. 43.)

Dr. Berg has never seen death ensue as an *immediate* consequence of aphthæ. Out of more than 600 post-mortem examinations of children, he has never observed *attached* aphthæ further than the cardiac orifice of the stomach, nor has he ever detected them in the air-passages.

Aphthæ can occur as a complication of almost all the diseases of childhood, and their growth is then favoured by the general deterioration of the fluids produced by these different disorders.

Our author considers the employment of the microscope to be the only certain mode of deciding on the presence of thrush, as there are many occasions where the nature of the exudation is doubtful, as for instance, where it is coloured by blood, or by pus from ulcerations or by dark ichor from gangrenous sores.

*Causes of the disease.* The theory of a "generatio æquivoca" to explain the immediate cause of the malady, cannot, Dr. Berg thinks, be adopted, as long as the possibility exists of the disease being otherwise conveyed. Dr. Berg suggests that the active principle of the sporules continues to exist after the crusts have been dried and pulverized, and on being again placed in favorable circumstances they will vegetate, in the same way as yeast when dried and then exposed to moisture, will still produce fermentation. This he has verified by ingenious experiments the details of which are too long for insertion here. From the consideration of this interesting point our author passes to the examination of those influences which eminently predispose the mucous membrane of the infant to the reception and increase of this disease.

*The predisposing causes* are of two kinds, mechanical and bio-chemical. The former require but a very brief notice, as they are only those which favour the localization of the sporules upon the mucous membrane of the mouth, &c. To the latter are referred the chemical constitution of the saliva and of the secretions of the mucous membrane, the presence of starch or of sugar or of other substances which might be converted into lactic acid, the presence of albumen or of any combination of protein containing azote, all of which more or less favour the growth of the sporules.

The first of the vital causes, which our author takes into consideration, is age. Aphthæ are not exclusively confined to the tender years of infancy, they are likewise found in childhood, and in maturer years, while in old age they again become more frequent. Dr. Berg observes that the somnolence, which is natural to very young children, is seen again in the aged, when suffering from other maladies, and he regards this symptom as a predisposing cause of aphthæ, from the mouth being left, for so long a period together, in a state of comparative repose. We confess that we do not ourselves attach much importance to this as a cause of aphthæ, but should the theory of Dr. Berg in regard to the true nature of this disease be the true one, it will of course acquire greater weight.

In considering the bio-chemical causes of aphthæ in children we must remember that the food of the child consists almost exclusively of fluids or solids containing sugar and starch, or albumen and casein, all of which the preceding experiments of Dr. Berg have proved to be eminently favorable to the growth of the parasite.

As regards *climate*, the warmer regions of the globe seem, as might naturally be expected, to be the most favorable to the production and growth of aphthæ, and they appear according to Auvity and Billard to be more frequent in summer than in the colder seasons of the year.

*Deficient ventilation* and want of cleanliness are acknowledged by all to be a powerful predisposing cause of aphthæ. A close, confined atmosphere is eminently favorable to the growth of a low type of vegetable life, while the unfavorable influence thus exercised on the health of the child, will render it more liable to all kinds of disease.

*Food and feeding.* The nature of the infant's food, and likewise the mode in which nourishment is given to it, is most carefully considered by our author. If the milk be of bad quality, it must necessarily impair the health of the infant, more or less, but Dr. Berg denies the existence of any peculiar acridity in the milk of some individuals, as has been asserted and believed by the public, and by many medical men from the earliest ages to the present time. It cannot however, as he observes, be denied, that the milk occasionally undergoes such changes as to render it unfit for the nourishment of the child, but the real nature of these alterations is as yet in a great measure unknown.

Much influence is ascribed by our author to the operation of artificial food in favouring the growth of aphthæ. It is not only that such food contains an abundance of starch and sugar, but also, that in many cases it may undergo a partial chemical alteration and decomposition before it is received by the infant. Dr. Berg does not regard the practice of feeding the infant with the sucking-bottle, as injurious, but it is necessary, he observes, to be careful that the milk flows freely through the artificial nipple appended to the vessel, as otherwise he thinks that the mucous membrane of the mouth might possibly be injured by the child's strenuous efforts to extract its food. Should the opening however be too free, then the mouth would become filled to excess with food, and this remaining more or less in that cavity, would produce injurious effects. These hints may to some appear trivial, and fit only for the consideration of mothers and nurses; but we think otherwise, and regard them as proofs of the just care and attention which Dr. Berg has bestowed on every portion of his subject.

Altered health, from whatever cause, undoubtedly predisposes children to this disease, by either producing a tendency to prolonged sleep, which eminently, in our author's opinion, favours the growth of the parasite, or in other cases by so altering the character of the secretions from the mucous membrane of the mouth as to render them important agents in augmenting the disorder. Dr. Berg does not agree with Andral and Gavarret in regarding thrush as a product of an excessive formation of albumen; he believes it to result rather from a reaction between the albumen and an acid.

*Contagion.* Our author adopts to the fullest extent the doctrine of the contagious nature of the malady which he describes. He believes that thrush may be conveyed from one patient to another by sporules, or fragments of sporules, in the dried state, floating in the atmosphere, but that it still more frequently is propagated by the bottles from which children with thrush have been fed, or by the nipple, especially where, as in many hospitals, two children are suckled by one nurse. To decide the question of contagion our author made numerous experiments, and the results were uniformly favorable to his previous ideas. The experiments were made by applying aphthous crusts to the mucous membrane of the mouths of perfectly healthy children, and in every instance thrush was rapidly produced. We cannot, however, call these trials in one respect perfectly successful, for they occasioned great suffering to the children and so materially interfered with the health of one of the little patients that it died afterwards of bronchitis and pneumonia. Although the death of this child cannot directly be referred to the experiments made upon it, we confess we should not ourselves feel perfectly at ease were such a result to ensue in our own practice. It is, however, but justice to our author to state, that after this untoward event he abstained from further researches of the kind.

*Prevalence of the disease.* Dr. Berg has spared no pains to make himself personally acquainted with the disease in different parts of Europe. Like many of his Scandinavian brethren, he has been an assiduous student in foreign schools. He has visited the foundling hospitals of Germany, Italy, and France, and has carefully perused the medical works relating to those countries he could not himself reach.

He does not regard the seasons of the year as having much influence on the prevalence of thrush. From 1842 to 1845, 733 patients affected with this disease were treated by him in the children's hospital in Stockholm. Of these 231 cases occurred in January, February, and March; 190 from April to June; 136 from July to September; and 176 from October to December; consequently there were 407 cases during the winter months, and 326 during those of summer. This proportion, he observes, would militate against the doctrine of the disease being more liable to occur in summer, but the fact is, that during the arctic winter of that high latitude, the apartments of the hospital are badly ventilated, and are kept at a high temperature by stoves, while the air is naturally less pure and less frequently renewed than in summer.

The fifth day after birth is the earliest period at which Dr. Berg has seen an infant affected with thrush.

One third of the children received into the hospital at Stockholm are attacked with the disease.

Relapses are not uncommon,—a previous attack does not seem to shield the patient from a return of the malady.

*Danger.* Much difference of opinion prevails in Stockholm as elsewhere as to the degree of danger incurred by patients labouring under this disease. Dr. Berg explains this by the fact, that these opposite opinions have been gathered under totally different circumstances. Those physicians who have observed thrush in private practice, or in lying-in hospitals, where children are suckled by their mothers, or by healthy nurses, are, he remarks, fully justified in asserting that thrush is a comparatively mild and insignificant disease. But where the malady is studied in large foundling hospitals, where the children are mostly brought up by hand, it assumes a much more fatal and malignant character, and fully bears out the assertion of the opposite party, that few disorders are more perilous to the infant. The more the disease is spread over the mucous membrane, the longer it resists treatment, the greater is the danger, especially if the vegetations be abundant and large, and if the whole assumes a yellow or dark brown hue.

*Prophylaxis.* The pages devoted to the prophylaxis of the disease are among the most interesting in the book. Dr. Berg insists strongly on the great advantages of cleanliness and of separation of diseased from healthy children. Without these all other precautions will be nearly useless. Were it possible, observes our author, to discover some chemical preparation which would effectually check the growth of the parasite, we might then regard the malady as one always within the power of medicine to heal. Perhaps the nitrate of silver in a solution of eight grains to the ounce of distilled water may effectually prevent the increase of the malady. We ourselves know that it constantly checks its further progress, and we may perhaps hope that if it were applied in good time to the mucous membrane it would effectually arrest the tendency to the increase of the vegetation.

*Treatment.* As the true nature of thrush has been an object of controversy, so the treatment of this disease has varied according to the doctrines adopted regarding its character. Some of the ancient and many too of the modern writers maintain the malady to be almost entirely local, and under this impression apply their remedies directly to the seat of the disease, while others, deriving it solely from milk of a bad quality supplied by the nurse or mother, restrict their treatment entirely to the latter, giving medicines to improve the quality of the milk, without any direct remedial interference with the child.

Dr. Berg steers a middle course between these opposite opinions, and while he bestows the utmost attention on the milk of the nurse or mother, he states that local treatment can never be entirely neglected; nay, that in many cases it forms the most important part of the cure. The variations in the character of the milk should be carefully studied, both chemically and with the microscope; aids to medical investigation which were formerly unknown, and even now are but imperfectly appreciated by the great mass of practitioners.

*General Treatment.* In the treatment of thrush, Dr. Berg observes, it is necessary to make a distinction between simple aphthæ or thrush occurring in an otherwise healthy child, and those forms of the disease which attend on other maladies. In simple thrush he regards general treatment as unnecessary; local applications will usually cure the disorder. Even when gastric affections supervene, a perseverance in the mere local treatment is often all that is required. But when the digestive organs become seriously affected, general remedies must also be had recourse to, and as these affections often remain after the aphthæ have disappeared, constitutional treatment should be persevered in till the health is completely restored.

Our readers will remember that Dr. Berg considers the disorder of the digestive functions in thrush to arise mainly from a superabundant formation of lactic, butyric, acetic, and carbonic acids, and consequently the alkalies and their carbonates are strongly recommended to neutralize the excess of acid. Of these he gives the preference to soda; but should an excessive development of gas ensue from its employment, he advises us to have recourse to "aqua calcis" and "magnesia usta," as a change of remedies is often urgently required.

Emetics are indicated when there is reason to suspect that the aphthous crusts have been swallowed to a great extent by the patient, and that they are acting injuriously on the contents of the stomach. When the child vomits spontaneously coagulated or uncoagulated milk or mucus, an emetic will have the most favorable effect. The quantity of acid generated in the intestinal canal causes an excessive formation of casein, and particles of a cheesy appearance are then passed in abundance by stool. In such cases purgatives are extremely useful in clearing the intestinal canal. For this purpose castor oil answers best, but when the bowels become slow, and much mucus is secreted from them, rhubarb and jalap should be exhibited. Calomel has very seldom been employed by Dr. Berg as a purgative in thrush.

To relieve the colicky pains and to obviate the tendency to intussusception so frequent in infants, Dr. Berg has generally employed antispasmodics and sedatives. The favorite preparation of this kind with our author is the *liq. succin. ammon.*, given either by the mouth, or in the form of an enema. In the majority of the milder cases, he has contented himself with administering a little of the *pulv. rhei* and magnesia. Dr. Berg warns us against giving any medicines in sugar to children suffering from aphthæ. He believes it to be an injurious practice, as the sugar being converted into lactic acid, will favour the increase of the parasitic vegetation.

*Local Treatment.* After a brief survey of the local remedies praised by the earlier writers, some of which are curious indeed, he observes that the local applications should be of such a nature as not to injure the epithelium on which the parasite is seated. "There is a period in the disease," he remarks, "when the aphthous crusts fall off of their own accord, and little more is then required than slightly pencilling or rubbing the affected part with a soft brush dipped in water." Dr. Berg, however, strongly advises us not to wait for this time, but, from the very beginning of the disorder to use chemical and mechanical remedies to destroy the parasite or to hinder its further extension. We cannot, however, expect to remove by direct mechanical interference the fibres of the parasitic growth, which have been shown by Dr. Berg to be so intimately interwoven with the uppermost layers of the epithelium. Our author, therefore, advises that in this stage we should have recourse to those remedies that have a gently solvent and macerating action on the epithelium. Among these he enumerates the alkaline carbonates, and also borax, while he considers the caustic alkalies to be more active in this regard, but, at the same time, less safe in their application. He has also tried solutions of alum, but not with very favorable results. The solutions of corrosive sublimate recommended by Columbier and Eisenmann are, he thinks, too perilous in their application to be ordinarily prescribed. *A certain quantity of acid* appears, from our author's researches, to be requisite for the growth of the parasite, while its increase is hindered by *an excess of acid*, and therefore the stronger mineral acids could not be used to arrest the disease, were not their local action too severe upon those parts of the mucous membrane that are still in a healthy condition. Alkaline remedies, consequently, are Dr. Berg's especial favorites. They counteract the excess of acidity so favorable to the growth of thrush, and, at the same time, soften the epithelium and aid in the throwing off of the aphthous crusts. An excess of alkali in any menstruum has been proved by Dr. Berg's experiments to arrest the increase of the parasite, and when administered to the child, alkalies probably counteract the excessive formation of lactic acid on the mucous membrane, though Dr. Berg does not positively state that they exercise a direct influence on the parasitic growth. Our author uses for this purpose solutions of subcarbonate of soda and borax, varying their strength according to the necessities of the case. The local treatment does not however end here. The constant application of alkaline solutions tends, according to our author, to diminish the thickness of the epithelial layers by their macerating and solvent properties, and thus the blood, he suspects, comes more immediately into contact with the fluids in the mouth; and he suggests that albumen may in this manner escape and become mixed with mucus; and when it comes in contact with lactic acid it will form one of the most favorable menstrua for the parasitic growth. It is in this way that Dr. Berg explains the frequency of relapses after alkaline treatment has been long employed, and he accordingly advises us then to adopt other remedies, especially astringents and the mineral acids. It is at this period too that nitrate of silver is so useful; and we ourselves have frequently administered it in the proportion of three or four grains to the ℥j of *syrup. rheados*, though perhaps the *infus. salviæ* or some other mucilaginous preparation containing less sugar would be a more appropriate vehiculum.

Finally, Dr. Berg opposes the doctrine of the older writers that thrush is usually followed by an eruption on the skin, and that this is to be regarded as the same exanthematous process transported to the external integument. Our readers will be aware that the exanthematous nature of aphthæ is entirely denied by our author, and in this respect we fully agree with him. The eruption in question is, as Dr. Berg observes, chiefly confined to the nates and the adjacent parts, which in young children are constantly wetted by urine, or are in contact with the fæces. When the secretions from the bowels become disordered and of an acrid character, they will necessarily irritate and inflame the neighbourhood of the anus, and in this way produce what by the vulgar is still regarded as the external and subsequent appearance of thrush. Cleanliness and attention to changing the linen is therefore of the utmost importance in preventing these sequelæ.

The second portion of Dr. Berg's Essay presents us with a nearly complete historical analysis of the descriptions of thrush, from the earliest ages down to the present time. We say nearly a complete analysis, for our author is candid enough to give a full list of those works which he has not been able to consult: these however, are comparatively unimportant, and while the former and purely original part of his Essay is a lasting monument of his great intelligence and unwearied zeal and of his scientific acquirements, we must give due praise also to our author for his diligence in collecting so large a body of information on the subject of aphthæ as is presented by the historical division of his work. His criticisms too on the researches of others are mild and temperate; he is no blind adorer of his favorite theory, for where experiments have turned out unfavorably to his preconceived opinions, he has recorded them as candidly as those which were successful. Dr. Berg has avoided the too common error of the present day in not hurrying with his newly formed doctrines to the press, fearful lest some more fortunate competitor might snatch the anticipated laurels from his brow. He has calmly and steadily pursued his experiments and matured his doctrines, ere he produced them to the medical world; and we trust that he will continue his researches, and extend them to other branches of infantile disease. Such a modest little volume as that now before us will be readily perused by the student who would shrink from a more extended work, while the high character of its contents will ensure for it a cordial reception from the medical world.

In the matter of printing and paper, the little book is superior to many of the works that have emanated from the Swedish press, while the errors are remarkably few, though quotations from seven or eight different languages are given in the historical portion. The Swedish school already occupies a favorable position in medical science, and we are convinced that this little volume will add to its reputation.

From the date of the publication of this monograph, the name of Dr. Fr. Th. Berg must take its permanent place in the list of original observers who have had the honour and good fortune of conferring a positive benefit on medical science.

---

